# HIV eradication: virological chances and clinical perspectives

**DOI:** 10.7448/IAS.15.6.18067

**Published:** 2012-11-11

**Authors:** C Perno

**Affiliations:** University of Rome ‘Tor Vergata’, Rome, Italy

## Abstract

Once a retrovirus infects a eukaryotic cell and integrates within chromosomal DNA, it becomes part of its genome and can be activated/transcribed/translated to produce viral proteins and/or new viral particles. Over the years, some of these retroviruses may lose their pathogenicity and become adapted to the new host. Under these conditions, retroviruses can paradoxically ameliorate the functional portfolio of the infected cell, thus potentially increasing its functionality and/or chances of survival in a difficult environment. Individuals whose cells are infected by retroviruses have acquired, in the last millions of years, innovative functions that, once transmitted to the new generation through germinal cells, have become essential for their homeostasis, or even for their survival. This is the case of some endogenous retroviruses, whose products are mandatory for the proper vascularization of human placenta. To our knowledge, there are no natural means able to selectively eliminate retroviral genes from an infected cell or an individual. Therefore, the chances of getting naturally cured by retroviruses, once infection is set and viral genomes are spread into the body, are minimal or absent. HIV is a retrovirus that behaves as all other retroviruses that interacted with humans in the past millennia. Its fate is to remain forever within the infected body. For these reasons, the chances of getting rid of HIV infection and being biologically cured (that is, eliminating all viral genomes from the body) are very limited if we consider current knowledge, biotechnology and available medical tools (yet it cannot be fully excluded in very peculiar cases). The option offered by the so-called “functional cure” is different. In this case, medical manipulation may create conditions whereby viral genomes, decreased in number and function by proper therapies/vaccines, are no longer able to harm the host for an indefinite period of time. Patients do remain infected, but viral replicative cycles are absent, and progression of the disease is interrupted. This latter clinical approach may be suitable, and this is where clinical research is directing its efforts. If achievable, infected persons should cope with the virus and keep it under control for decades, without support of chronic antiviral therapy. In conclusion, the proper knowledge of the biological characteristics of HIV helps in selecting the best strategies aimed at obtaining the maximum achievable clinical result.

